# Supplementary material for: Unveiling Monoterpene Biosynthesis in Taiwania cryptomerioides via Functional Characterization
Source: Plants (Basel). 2021 Nov 8;10(11):2404. doi: 10.3390/plants10112404 (PMC8625326; doi:10.3390/plants10112404)
Supplement: Supplementary file 1 [file plants-10-02404-s001.zip › plants-1417452-supplementary.pdf]

Supplementary information

Title

Unveiling the monoterpene biosynthesis in *Taiwania cryptomerioides* via functional characterization

Authors

Li-Ting Ma, Pi-Ling Liu, Yang-Tui Cheng, Tz-Fan Shiu and Fang-Hua Chu

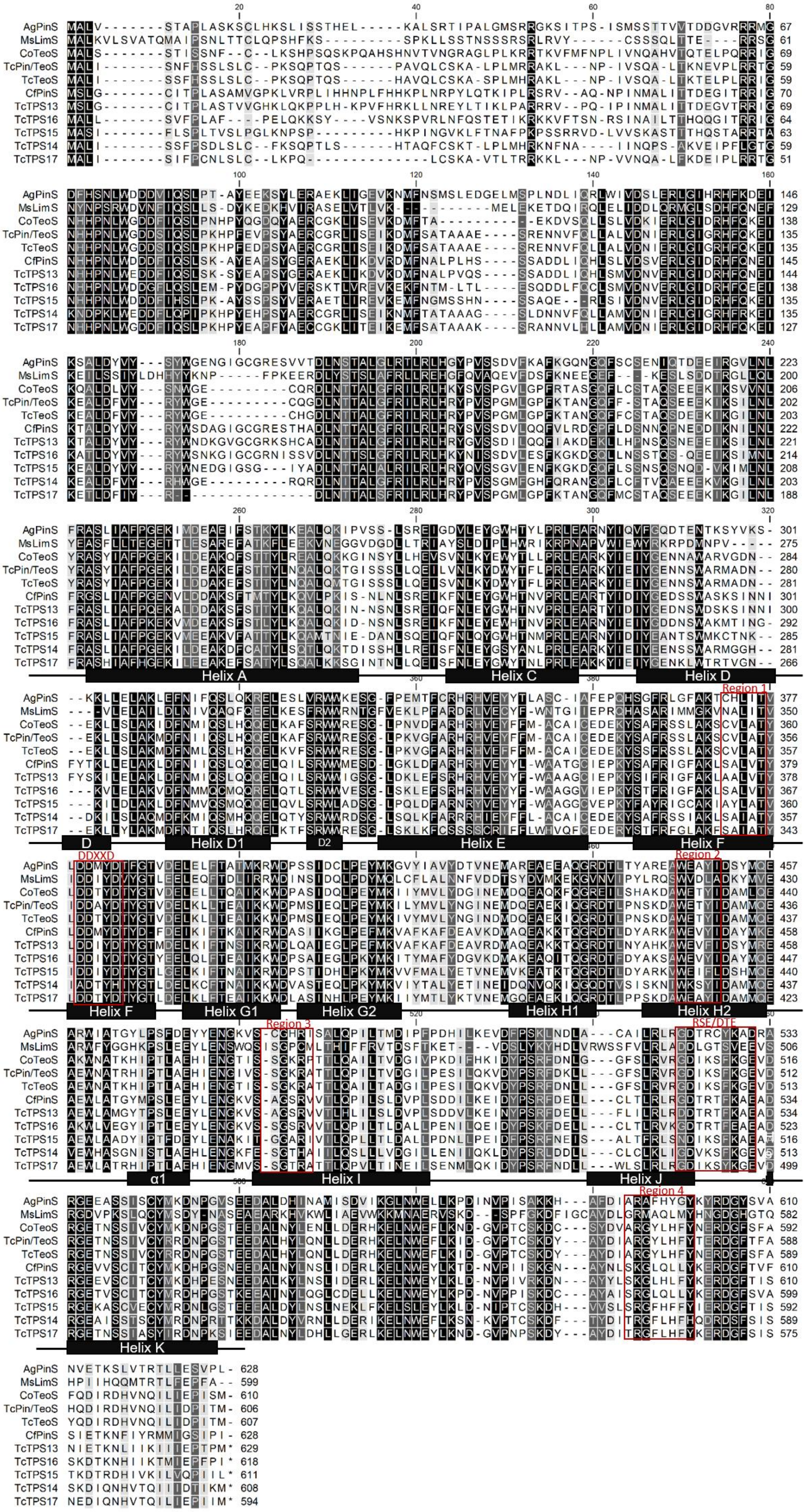

AgPinS
MsLimS
CoTeoS
TcPin/TeoS
TcTeoS
CfPinS
TcTPS13
TcTPS16
TcTPS15
TcTPS14
TcTPS17
Helix A
Helix C
Helix D
Region 1
D
Helix D1
D2
Helix E
Helix F
DDXXD
Region 2
Helix G1
Helix G2
Helix H1
Helix H2
Region 3
RSE/DTE
α1
Helix I
Helix J
Region 4
Helix K

Figure S1. The full alignment of *Tc*TPS13~16 and selected monoterpene synthases. The black bars represent the location of helixes. Red boxes represent plasticity residues and metal ion binding site DDxxDD and NSE/DTE motif.

Table S1 Primers used in this study.

Primer name	Sequence
*Tc*TPS15_5R1	CTGGAAGAAGGTTGTCAGGAAGAAGTGC
*Tc*TPS15_5R2	GAGCTCACCAAGTGTCCCATATGTGTC
*Tc*TPS15_5R3	CACCTGAAGAGACTTGGTATCTGTGCAG
*Tc*TPS15_full_F	CACTTGCCGAGCATGGCTTCCATC
*Tc*TPS15_full_R	GATAATCATTGAAGTACTAAAACTATAATATAATAGGTTG
*Tc*TPS15_NotI_F	ATAAGATTGCGGCCGCATGGCTTCCATCTTCCTTTC
*Tc*TPS15_EcoRI_R	GGAATTCCTATAATATAATAGGTTGAACTAGGATTTTCAC
*Tc*TPS13_F	CGAGCTTCACTTATAGCGTTTCCTCAGGAG
*Tc*TPS13_R	GTTGTTGTAGTGACTGAATCATATTAAAGTC
*Tc*TPS13_3R1	CGAGCTTCACTTATAGCGTTTCCTCAGGAG
*Tc*TPS13_3R2	CTCTCAAGATGGTGGATAGGATCTGGTTTG
*Tc*TPS13_5R1	GCTCATCCATAGTTCCATAAGTGTC
*Tc*TPS13_5R2	CAAACCAGATCCTATCCACCATCTTGAGAG
*Tc*TPS13_5R3	CTTCCAATCTAGGTACATTGGTGTGCCAAC
*Tc*TPS13_F2	GAGCCATTATGTCTCTCGGATGCATTACG
*Tc*TPS13_R2	GATTAGCCAAGTCACATTGGAGTTGGCTC
*Tc*TPS13_BamHI_F	CGGGATCCCGACGCATTGGCAATCATCACCCTAAC
*Tc*TPS13_XhoI_R	CCGCTCGAGCATTGGAGTTGGCTCAATTATTATCTTGATGATAAG
*Tc*TPS14_F	CAAATGGGCAGTTCTTGTGCTTCACAG
*Tc*TPS14_R	GAAGCTGTTCAGTAGATGCTACATCCCAC
*Tc*TPS14_3R1	GAGTATGGCTCGTACGCCAATCTTCC
*Tc*TPS14_3R2	CCTACTCCAGATGGTGGAGGGATTCTG
*Tc*TPS14_5R1	GCATGCCACTCAACTTCTTGCATCATG
*Tc*TPS14_5R2	GAGTAGGTTTGAAGCTCCTGCTGATGC
*Tc*TPS14_5R3	GAGCTGATATCAGTCTTCTGCAGAGTTTGAC
*Tc*TPS14_F2	CATACCATACCTTGCAATGGCTCTTATCTCCAG
*Tc*TPS14_R2	CATAGCTGAATGACACCTAGATTTAGATCATTAC
*Tc*TPS14_BamHI_F	CGGGATCCCTGGGCACGGGCAAAAATGATCCAAAATTG
*Tc*TPS14_XhoI_R	CCGCTCGAGCATTTTAATGGTGTCAATAATAATTTGGG
*Tc*TPS16_F	CCATCACCCTAACTTGTGGGATGATGG
*Tc*TPS16_R	CCTCGTGATATTGCATAAGCACAATCCTTG
*Tc*TPS16_3R1	CGAAATCACTCAAGGCCGAGACACATTCG
*Tc*TPS16_3R2	GAGGCTGAAGCTGAACGCGGAGAAATAG
*Tc*TPS16_5R1	GCAAAGTCACAACACGTGAACCACTACTGAC
*Tc*TPS16_5R2	CGTTGTTGCATGCACTGCATCATGTTGAAG
*Tc*TPS16_5R3	CATCCGATGCCTTTGTTGCTCCAATACCTG

*Tc*TPS16_F2	GTTAGGATGGCTTTGCTTTCCGTATTCC
*Tc*TPS16_R2	GAGTCATATTGGAAAAGGTTCAATCATGGTTTTGATG
*Tc*TPS16_SalI_F	ACGCGTCGACAAGTATCTAACAAGTCTCCCGTTAGATTAAATTTTC
*Tc*TPS16_XhoI_R	CCGCTCGAGTATTGGAAAAGGTTCAATCATGGTTTTGATG
*Tc*TPS17_3R1	ATGAGGCTCCTTTCTATGCCAAATGTTGTG
*Tc*TPS17_3R2	CAGTGCAACTGCAGCTGCTAAATCAC
*Tc*TPS17_5R1	TGTAGAATACCTCTCATCCTCAC
*Tc*TPS17_5R2	GATGCAATGACTGAATTGTGTTGAAGTC
*Tc*TPS17_5R3	GATTGGTGTACCAGCCATACTCCAGATTG
*Tc*TPS17_5R4	CTCTGATTGAGCCGTGGAGCACATG
*Tc*TPS17_5R5	GTCGATCAATTCCAAGACGTTCAATGTTATC
*Tc*TPS17_F	CGTTGCTTGCAATGGCTCTTATCTCCA
*Tc*TPS17_R	CTATATTGAGGGGTTACATGATTATAGGTTCAATGAG

Table S2. The protein sequences of terpene synthases used in phylogenetic analysis

Names	Accession #	Species	Description
*Aa*CarS	AAL79181	*Artemisia annua*	β-Caryophyllene synthase
*Aa*PinS	AAK58723	*A. annua*	β-Pinene synthase
*Ag*AS	AAB05407	*Abies grandis*	Abietadiene cyclase
*Ag*BisS	AAC24192	*A. grandis*	*E*-α-Bisabolene synthase
*Ag*HumS	AAC05728	*A. grandis*	γ-Humulene synthase
*Ag*LimS	AAB70907	*A. grandis*	(-)-4*S*-Limonene synthase
*Ag*MyrS	AAB71084	*A. grandis*	Myrcene synthase
*Ag*PinS	AAB71085	*A. grandis*	Pinene synthase
*Ag*SelS	AAC05727	*A. grandis*	δ-Selinene synthase
*Ag*TerS	AAF61454	*A. grandis*	Terpinolene synthase
*Am*OciS	AAO42614	*Antirrhinum majus*	(*E*)-β-Ocimene synthase
*At*Car/HumS	AAO85539	*Arabidopsis thaliana*	β-Caryophyllene/α-humulene synthase
*At*Ks	Q9SAK2	*A. thaliana*	*ent*-Kaurene synthase
*At*LinS	AAO85533	*A. thaliana*	Linalool synthase
*Ca*CPS	ACQ99373	*Coffea arabica*	Copalyl diphosphate synthase
*Cf*CadS	AFJ23663	*Chamaecyparis formosensis*	β-Cadinene synthase
*Cf*PinS	ABW80964	*C. formosensis*	α-Pinene synthase
*Cl*TerS	AAM53943	*Citrus limon*	γ-Terpinene synthase
*Co*TerS	BAI53108	*Chamaecyparis obtusa*	Terpinolene synthase
*Ga*CadS	AAD51718	*Gossypium arboreum*	δ-Cadinene sythase
*Gb*BisS	AIU94290	*Ginkgo biloba*	α-Bisabolene synthase
*Gb*FarS	AIU94289	*G. biloba*	(*E,E*) Farnesol synthase
*Gb*LS	AAL09965	*G. biloba*	Levopimaradiene synthase
*Md*Fars	AAO22848	*Malus domestica*	(*E,E*)-α-Farnesene synthase
*Mg*CubS	ACC66281	*Magnolia grandiflora*	β-Cubebene synthase
*Pa*FarS	AAS47697	*Picea. abies*	*E,E*-α-Farnesene synthase
*Pa*LAS	AAS47691	*P. abies*	Levopimaradiene/abietadiene synthase
*Pa*LimS	AAS47694	*P. abies*	(-)-Limonene synthase
*Pa*LinS	AAS47693	*P. abies*	(-)-Linalool synthase
*Pa*LonS	AAS47695	*P. abies*	Longifolene synthase
*Pa*MyrS	AAS47696	*P. abies*	Myrcene synthase
*Pa*PinS	AAS47692	*P. abies*	(-)-α/β-Pinene synthase
*Pg*CPS	ADB55707	*Picea. glauca*	*ent*-Copalyl diphosphate synthase
*Pg*KS	D2X8G1	*P. glauca*	*ent*-Kaurene synthase
*Pm*BisS	AAX07266	*Pseudotsuga menziesii*	(*E*)-γ-Bisabolene synthase
*Pm*FarS	AAX07265	*P. menziesii*	(*E*)-β-Farnesene synthase

*Pp*CPS_KS	BAF61135	*Physcomitrella patens*	*ent*-Copalyl diphosphate/*ent*-kaurene synthase
*Ps*LimS	ABA86248	*Picea sitchensis*	(-)-Limonene synthase
*Ps*PinS	AAP72020	*P. sitchensis*	Pinene synthase
*Ptd*GerS	AAR99061	*Populus trichocarpa* x *deltoides*	(-)-Germacrene D synthase
*Pt*PinS	AAO61228	*Pinus taeda*	(+)-α-Pinene synthase
*Pt*TerS	AAO61227	*P. taeda*	α-Terpineol synthase
*Sl*CPS	BAA84918	*Solanum lycopersicum*	*ent*-Copalyl diphosphate synthase
*Sr*CPS	AAB87091	*Stevia rebaudiana*	Copalyl pyrophosphate synthase
*Sr*KS	AAD34294	*S. rebaudiana*	Kaurene synthase
*Tb*TaxS	AAC49310	*Taxus brevifolia*	Taxadiene synthase
*Tc*CPS2	AOG18229	*Taiwania cryptomerioides*	Labda-13-en-8-ol diphosphate synthase
*Tc*CPS3	AOG18237	*T. cryptomerioides*	*ent*-Copalyl diphosphate synthase
*Tc*CPS4	JN587309	*T. cryptomerioides*	(+)-copalyl diphosphate synthase
*Tc*KSL1	AOG18231	*T. cryptomerioides*	Biformene synthase
*Tc*KSL3	ADL14246	*T. cryptomerioides*	Levopimaradiene synthase
*Tc*Pin/TeoS	AIO10962	*T. cryptomerioides*	α-Pinene/terpinolene synthase
*Tc*TeoS	AIO10963	*T. cryptomerioides*	Terpinolene synthase
*Tc*TPS1	QGN65607	*T. cryptomerioides*	Zingiberene synthase
*Tc*TPS10	QHZ00913	*T. cryptomerioides*	Unknown function
*Tc*TPS11	QHZ00914	*T. cryptomerioides*	Unknown function
*Tc*TPS12	QHZ00915	*T. cryptomerioides*	δ-Cadinene/ Germacrene-4-ol synthase
*Tc*TPS2	QGN65608	*T. cryptomerioides*	Sesquiterpene synthase
*Tc*TPS3	QGN65609	*T. cryptomerioides*	δ-Cadinene synthase
*Tc*TPS4	QGN65610	*T. cryptomerioides*	Germacrene-4-ol synthase
*Tc*TPS5	QGN65611	*T. cryptomerioides*	Longifolene synthase
*Tc*TPS6	QGN65612	*T. cryptomerioides*	Cedrol synthase
*Tc*TPS7	QGN65613	*T. cryptomerioides*	Sesquiterpene synthase
*Tc*TPS8	QGN65614	*T. cryptomerioides*	Germacrene D synthase
*Tc*TPS9	QGN65615	*T. cryptomerioides*	Caryophyllene synthase
